# Use of open source monitoring hardware to improve the production of MOFs: using STA-16(Ni) as a case study

**DOI:** 10.1038/s41598-020-73780-z

**Published:** 2020-10-15

**Authors:** Felicity Massingberd-Mundy, Stephen Poulston, Stephen Bennett, Hamish Hei-Man Yeung, Timothy Johnson

**Affiliations:** 1 Johnson Matthey Technology Centre, Blount’s Court, Sonning Common, Reading, RG4 9NH UK; 2 Inorganic Chemistry Laboratory, University of Oxford, South Parks Road, Oxford, OX1 3QR UK; 3 Present Address: School of Chemistry, University of Birmingham, Edgbaston, Birmingham, B15 2TT UK

**Keywords:** Chemistry, Materials science

## Abstract

Affordable and readily available microelectronics are becoming prevalent in teaching laboratories however these useful and economic tools are not used widely in either academia or industry. Herein we report how a metal organic framework (MOF) synthetic route can be optimized using an in situ monitoring apparatus designed in-house on open source hardware for under $100. We demonstrate that the MOF can be produced at atmospheric pressure, an improvement over previous reports, but also with a reduction in reaction time of 93%. This improvement in reaction time was predicted after a single experiment using the monitoring kit showing how efficiencies in the lab can be gained with very little experimental and monetary overhead while minimising the resources used.

## Introduction

The increasing availability of affordable open source microelectronics presents research scientists with a unique opportunity to prototype instrumentation specifically designed around their research needs. Bespoke apparatus offers many advantages over commercial offerings—with flexibility, cost and ownership being key. A growing number of reports have demonstrated the use of these types of apparatus in education^1,2^, notably the ability to produce systems for students with disabilities^3,4^. Several reports have also demonstrated how such equipment can be used outside the teaching laboratories with the development of potentiometric detection^5^, scanning electrochemical microscopes^6^ and electrochemical pre-treatment apparatus^7^ all using inexpensive and open source hardware solutions.

One area of interest to materials research scientists is the ability to log data from a range of sensors to gain insight into a reaction mixture, in situ. Mercer et al*.* demonstrated that a cost-effective device could be produced using Internet of Things (IoT) connected microcontrollers that could log turbidity data^8^. While insight gained into the precipitation of NaCl from Na_2_S_2_O_3_ and HCl was useful in the context of chemical education, insight gained from this type of measurement may also be of interest to scientists studying novel systems.

Within our laboratory the production and optimization of metal organic frameworks is an important and active area^9^. MOFs are a possible disruptive innovation with applications including gas separation, catalysis, drug delivery and medical devices^10–13^. These materials are becoming a target for commercialization as their applications are continually demonstrated. While much work has focused on design^14^ and fundamental understanding^15^, commercialization of these preparation routes is not a trivial task. Poor solvent choice, low concentrations and the use of pressure vessels plague the literature. Optimization of preparation routes is key if a given material is to be of commercial relevance.

Time-resolved ex situ and in situ techniques have been used to investigate MOF crystallization mechanisms and reaction intermediates—key aspects of material scale-up^16^. Such techniques include X-ray diffraction, small-angle and wide-angle X-ray scattering (SAXS and WAXS), NMR, dynamic and static light scattering (DLS and SLS), and scanning and transmission electron microscopy (SEM and TEM). The downside to these techniques is that they are often expensive and complicated to use, requiring specific training.

Recently, Yeung et al*.* used in situ synchrotron XRD to monitor the formation of ZIF-8, shedding light on its complex crystallization mechanism^17^. In situ laboratory pH measurements and turbidity measurements, the latter performed using a low-cost Raspberry Pi-based apparatus, showed similar trends to XRD, suggesting that important insights could be gained from the use of cheaper consumer electronics alone.

**Figure 1:**
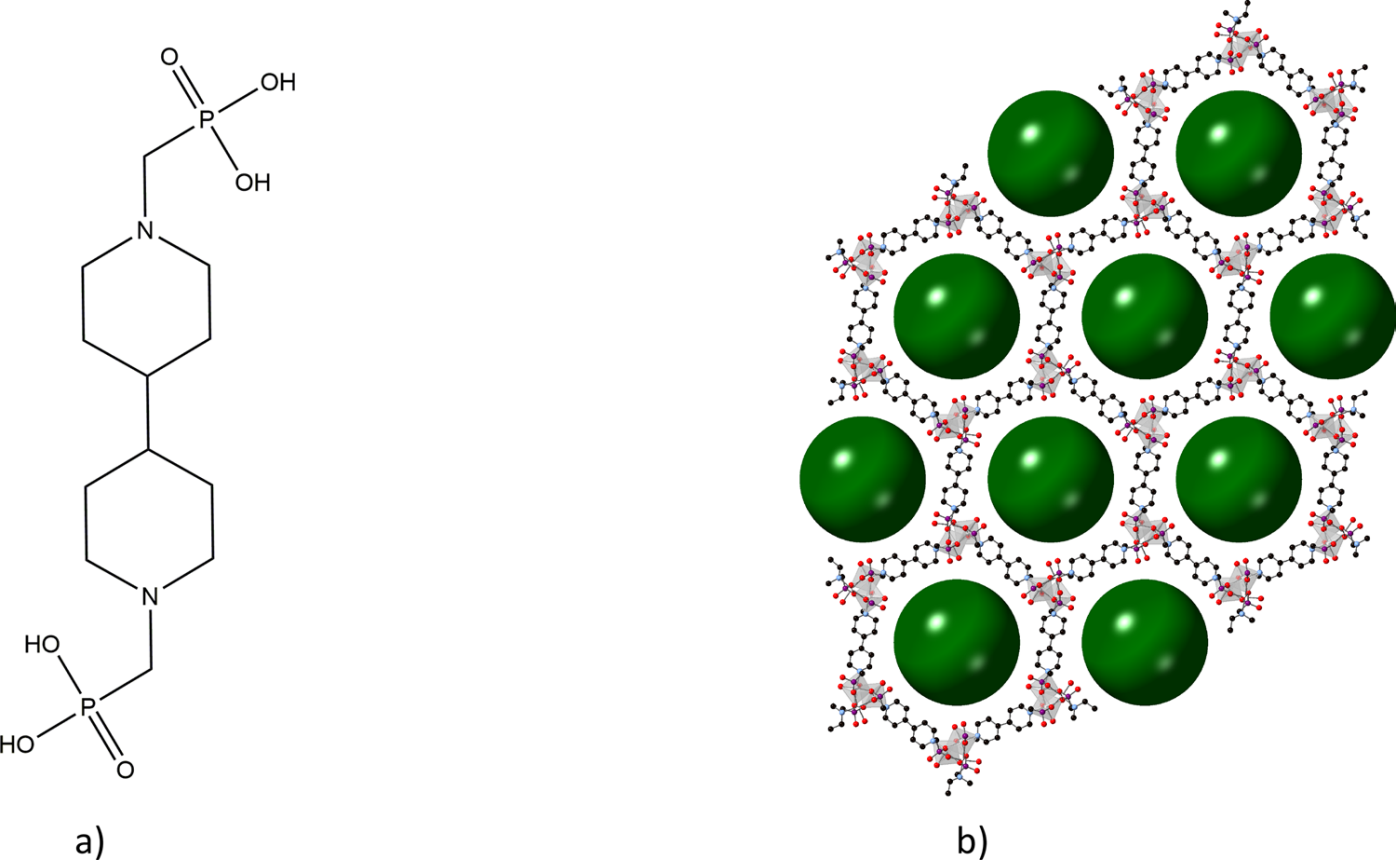
(**a**) STA-16 linker and (**b**) representation of the STA-16(Ni) framework. Black, light blue, red and purple spheres represent carbon, nitrogen, oxygen and phosphorous atoms respectively. Ni–O polyhedra are represented in light grey and green spheres (r = 2 nm) are used to illustrate the pore voids. Protons omitted for clarity.

To understand how in situ reaction monitoring could be used to specifically optimize MOF production routes, the STA-16(Ni) system was studied. This material consists of octahedra metal centers connected to the linker *N*,*N*′-4,4′-bipiperidinebis(methylenephosphonic acid), Fig. 1a, further referred to as STA-16 Linker.

STA-16 is an exceptional material due to its large pore size and diverse range of metal centers—with Co, Ni, Fe, Mn, and Mg reported^18,19^. A representation of the framework can be seen in Fig. 1b. The dehydrated forms of these materials have coordinatively unsaturated sites^18,20,21^, with the consequential Lewis acidity resulting in catalytic applications for these structures^19,22^. These MOFs have also been investigated for use in gas adsorption and separation^18,21,23^. The larger pores of the STA-16 structure make it suited to capturing larger molecules^20^. This makes the STA-16 frameworks particularly interesting for industrial exploration due to its potential use in a diverse range of applications. While this framework is of interest, its use is stymied by its production using pressure vessels and a reported reaction time of 72 h—limiting both space and time yields^20^. Ultimately, improving the reaction procedure for this material will signify a significant cost saving as well as a reduction in energy and resource usage. We report the design of a multi-parameter in situ monitoring apparatus constructed from open source hardware and its use in the optimization of the synthesis of STA-16(Ni). We find that the material can be produced with a reduction in reaction time of 93% over the reported procedure, without the use of pressure vessels. We believe this is the first example of an instrument (which is bespoke, produced on a budget and open source) being used to investigate and improve the production of a material of industrial relevance resulting in the delivery of quality material in a speedy and economic fashion.

## Methods

### Materials

Hydrobromic acid solution, phosphorous acid, 4,4′-bipiperidine dihydrochloride and nickel(II) acetate tetrahydrate were purchased from Alfa Aesar with formaldehyde and KOH obtained from Fisher Scientific. All chemicals were used without further purification.

### STA-16 linker

The STA-16 linker was produced using a modified version from past reports^20^. In brief, 4,4′-bipiperidine dihydrochloride (14.35 g, 0.06 mol) and phosphorous acid (12.8 g, 0.156 mol) were dissolved in distilled water (60 ml). To this hydrobromic acid solution (42 ml; 48 wt% aqueous solution) was added and the mixture was stirred until all the solids had dissolved. Formaldehyde (26 ml, 0.35 mol; 35 wt% aqueous solution) was added dropwise to the reaction mixture over 30 min and the solution was heated to 120 °C for 20 h. The reaction mixture was chilled at—22 °C overnight to facilitate full crystallization. The product was separated by vacuum filtration and washed with cold ethanol:water mixtures (90:10, 3 × 30 ml) prior to drying at 40 °C overnight in static air.

### STA-16(Ni) synthesis

STA-16(Ni) was produced at various scales with exact quantities for each reaction shown in Table 1. An example synthesis for Entry 1 is as follows. STA-16 Linker (5.18 g, 0.42 mmol) was added to H_2_O (250 ml). To this KOH (29.75 ml, 1.0 M) was added followed by nickel(II) acetate tetrahydrate (7 g, 0.82 mmol). The reaction mixture was aged at room temperature for 45 min before heating to 120 °C for 65.25 h—a total reaction time of 66 h. Once cooled the sample was separated by filtration and washed three times with 1 M KOH followed by two washings with water. The material was activated under vacuum at 120 °C overnight prior to further analysis.

**Table 1:** Reagent quantities to produce STA-16(Ni).

Entry	Linker (g)	H_2_O (ml)	1 M KOH (ml)	Ni acetate (g)
1	5.18	250	29.75	7
2	10.36	500	60	14
3	0.52	25	3	0.7

### ZIF-8 synthesis

The production of ZIF-8 was modified from previous reports^24^. In brief—solutions of Zn(NO_3_)_2_·6H_2_O (1.40 g, 4.7 mmol) in methanol (95 ml) and 2-methylimidazole (3.09 g, 37.6 mmol) in methanol (95 mL) were prepared. The in situ pH, turbidity and temperature probes were then placed in the 2-methylimidazole solution. Once the pH and turbidity had stabilized, the zinc nitrate solution was added under stirring. The resulting product was collected by centrifugation and the sediment was washed 3 times with methanol. The product was dried in air at room temperature overnight. The sample was activated at 50 °C overnight prior to characterization.

### Arduino® apparatus

**Figure 2:**
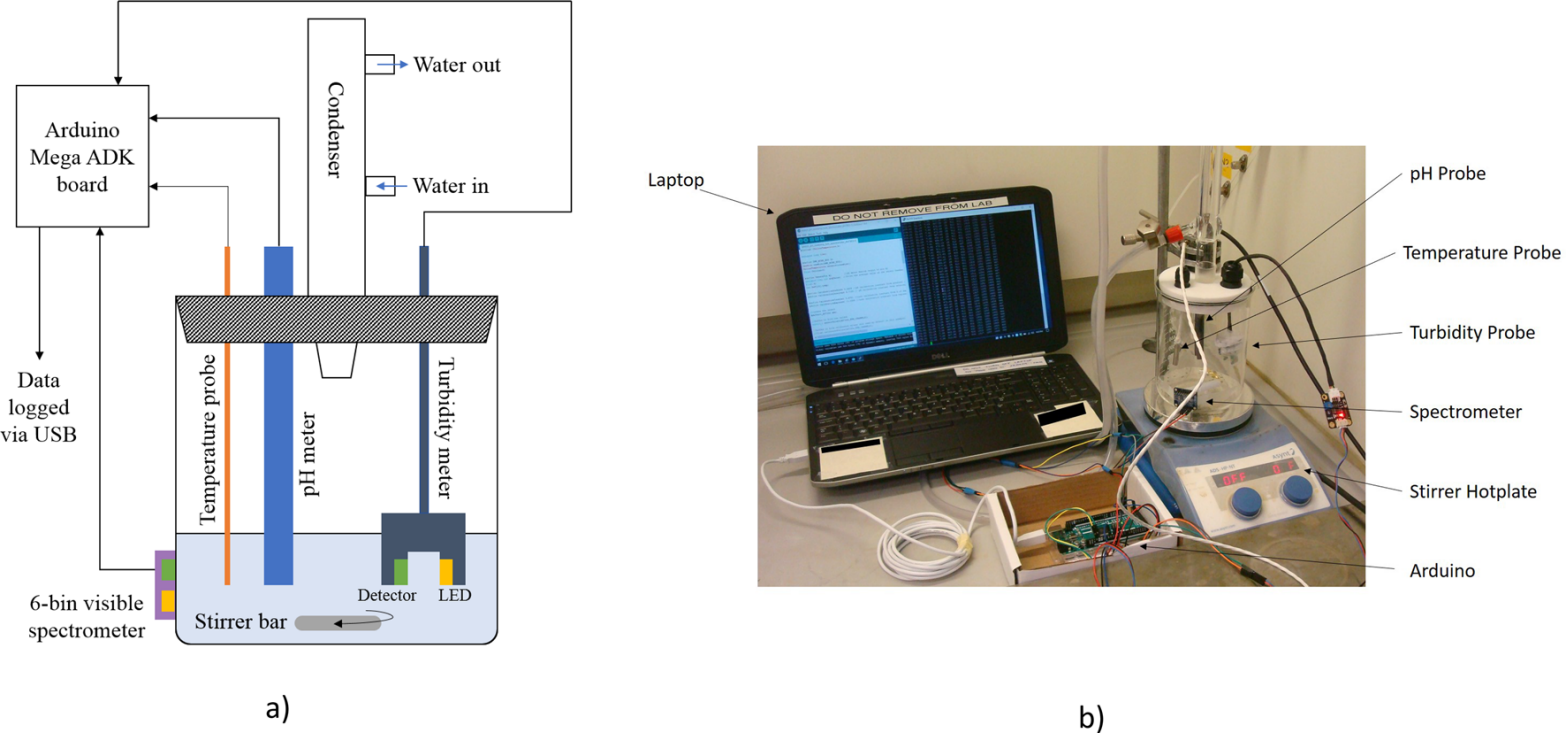
(**a**) Schematic representation and (**b**) a photograph of the Arduino® in situ monitoring apparatus.

Figure 2a shows a schematic representation of the in situ monitoring apparatus with Fig. 2b showing a photograph of the apparatus. The apparatus consists of an Arduino® Mega prototyping board with pH, turbidity and temperature sensors. These sensors are placed within the reaction media. A six-channel visible light spectrometer was also connected and secured to the outside of the reaction vessel. Color readings of the reaction mixture, along with temperature, turbidity and pH are all recorded by the Arduino® and stored on the attached PC.

Before each experiment the turbidity sensor was calibrated using Formazin turbidity standards of 1, 5, 10, 100, 1000, 2000, and 4000 Nephelometric Turbidity Units (NTUs), and the pH meter was calibrated against buffer solutions of pH 4.02, 7.00, 9.00, and 10.00. Calibration curves were produced by plotting the output of the turbidity meter against the turbidity of the standard, and the output of the pH meter against the pH of the buffer solution. An exponential fitting was used for the turbidity calibration curve. A linear fitting was used for the pH meter calibration as this produced the highest *R*^2^ value.

The temperature probe was calibrated against digital thermometer readings of a heated water sample and the readings of the probe were found to be accurate to ± 0.5 °C. The derived parameters from the turbidity calibration and pH calibration fittings were applied within the code for the Arduino® board to convert the analogue pH and turbidity outputs into readings in units of pH and NTUs respectively.

In situ visible spectroscopy measurements were recorded during the MOF syntheses. The spectrometer was secured to the outside of the glass reaction vessel. Luminance was measured in units of μW cm^−2^. A time-resolved spectrum was then produced by plotting the normalised luminance against time.

### XRD

Powder X-ray diffraction (PXRD) data were collected in reflection geometry using a Bruker AXS D8 diffractometer using Cu Kα radiation (λ = 1.5406 + 1.54439 Å) over the 5 < 2θ < 50° range in 0.02° steps. Refinement was performed using Topas^25^ with reflection profiles modelled using a fundamental parameters approach^26^ with reference data collected from NIST660 LaB_6_.

### Physisorption

Samples were subjected to analysis by N_2_ physisorption. Isotherms were collected on a TriStar II instrument with experiments being conducted at − 196 °C in N_2_. All samples were degassed at 120 °C for 12 h prior to analysis.

## Results and discussion

Previous reports of the STA-16(Ni) framework rely on the use of autogenous pressure to produce the framework. Initial work conducted for this study focused on producing the material using conditions that are more amenable to large scale production. To this end the preparation route was successfully conducted at atmospheric pressure under reflux conditions. Table 1 Entry 1 shows the quantities of reagents used for this reaction. The STA-16 linker required for these experiments was produced in accordance to literature reports^20^. The collected PXRD and subsequent Rietveld refinement can be seen in Supplementary Fig. S1 online. This shows a phase pure linker was obtained.

**Figure 3:**
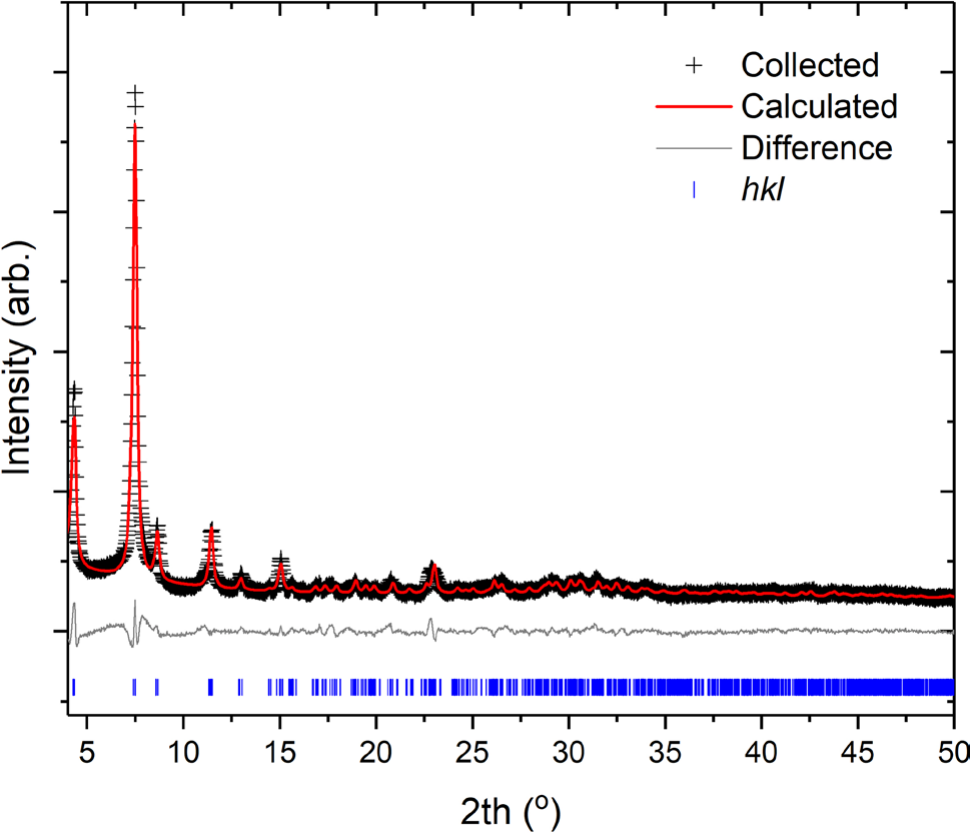
Collected PXRD pattern and subsequent Rietveld refinement for STA-16(Ni) produced at 62 h under reflux conditions (Entry 1).

Figure 3 shows the collected PXRD and subsequent Rietveld refinement demonstrating a phase pure STA-16(Ni) material has been produced. While this route is not optimized, with a yield of only 18.35%, it does demonstrate this material can be produced in an agreeable manner without the need for expensive pressure vessels with limited space time yields.

While demonstrating the material can be produced at atmospheric pressure under reflux conditions, a significant improvement over literature reports, the framework still required 66 h to produce. This is not acceptable if the material is to be produced commercially. For this reason, the preparation route was subjected to in situ reaction monitoring.

The bespoke reaction monitoring apparatus, shown in Fig. 2, was used to monitor the formation of the STA-16(Ni) framework. The key to this piece of novel apparatus is the ability to connect, log and analyze data from a range of different commercially available sensors. The apparatus can simultaneously monitor pH, turbidity, temperature and the visible spectrum of a reaction mixture. This approach means that, even when very little physical information is known about the system, the chance of observing a reaction critical process is high.

The code used to collect the in situ data is reproduced in Supplementary Fig. S2 online. The apparatus was produced and coded for under $100—with a parts list shown in Table 2. This makes it ideal for monitoring reactions of interest in the lab regardless of budget.

**Table 2:** Parts list for the in situ monitoring device.

Part	Manufacturer	Product code	Price (USD)
Turbidity probe	DFRobot	SEN0189	10
Thermocouple	Adafruit	DS18B20	7
6-Channel visible light sensor	Adafruit	AS7262	20
pH Probe	DFRobot	SEN0161	30
Arduino® mega	Arduino®	A000067	31

Pricing from manufacture at time of writing.

**Figure 4:**
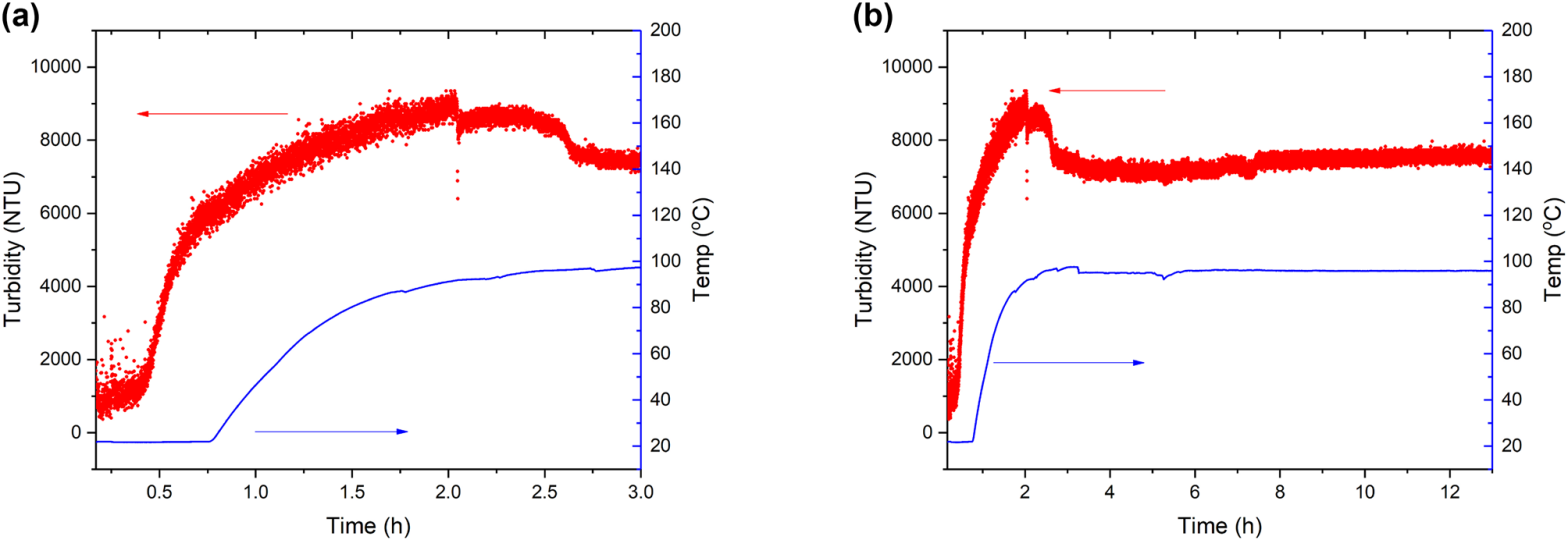
(**a**) Turbidity and temperature data obtained from the in situ reaction monitoring kit , and (**b**) the same data with an extended time axis.

**Figure 5:**
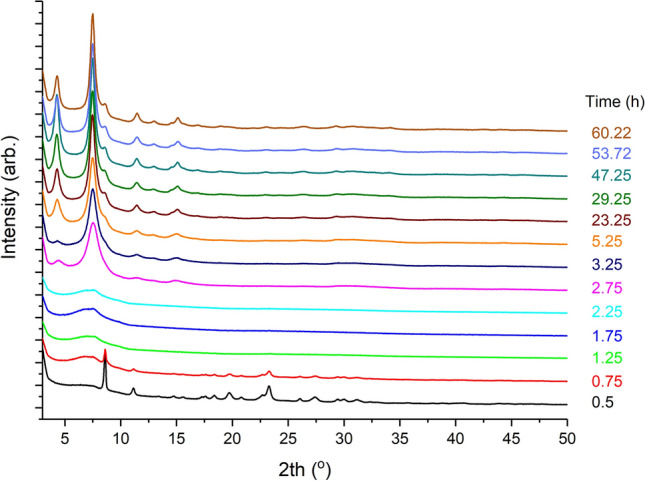
XRD patterns for the time-resolved samples removed from the STA-16(Ni) synthesis.

In situ turbidity data, Fig. 4, show clearly how, during the initial 45 min of aging, the reaction mixture becomes more turbid. Upon heating the reaction mixture reaches a maximum value of ~ 9000 NTU. After this a drop in turbidity is noted before stabilizing. This drop is likely due to a critical concentration of a metal-linker species producing large solid particles of MOF which reduce the turbidly of the overall solution. Additionally, pH and spectrometer data show similar responses. Supplementary Fig. S3 online shows data collected from the pH and spectrometer sensors during the reaction. The pH data is consistent with the formation of the MOF and the liberation of protons from the used linker- this effect reaches equilibrium after 5 h. Additionally, the spectrometer data show how changes can be observed in the sensor response during the aging and heating segments of the reaction. This is in line with empirical observations as the reaction mixture goes from colorless to green on addition of the Ni salt. This reaction mixture became a lighter green overtime, again supported by the collected data. In these data a plateau is also observed suggesting no further change occurs after the first 5 h. Combining pH and turbidity data suggest that the reaction has reached completion between 4 and 5 h, not 72 h as previously reported—an improvement of 93%. To determine if the reaction truly reached completion after 5 h, a reaction was conducted in which samples were removed at various times. Figure 5 shows the PXRD patterns obtained from this experiment. It can be seen how peaks indexable to the STA-16 linker can be observed up to 1.25 h. From 0.75 to 2.25 h a broad Bragg diffraction peak is observed at ~ 7° 2th, with significant diffraction from the STA-16 framework only being observed from 2.75 h onwards. At 5 h a well-defined STA-16 framework is observed with increasing reaction time not producing further diffraction information. These data also allow for further elucidation of the crystallization process for these materials. It can be seen how amorphous material is observed prior to 2.25 h with a peak indexable to STA-16 (Ni) observable after 2.75 h. It is within this time window that a drop-in turbidity is observed which is consistent with a critical concentration of metal and linker allowing for the formation of crystalline MOF.

**Figure 6:**
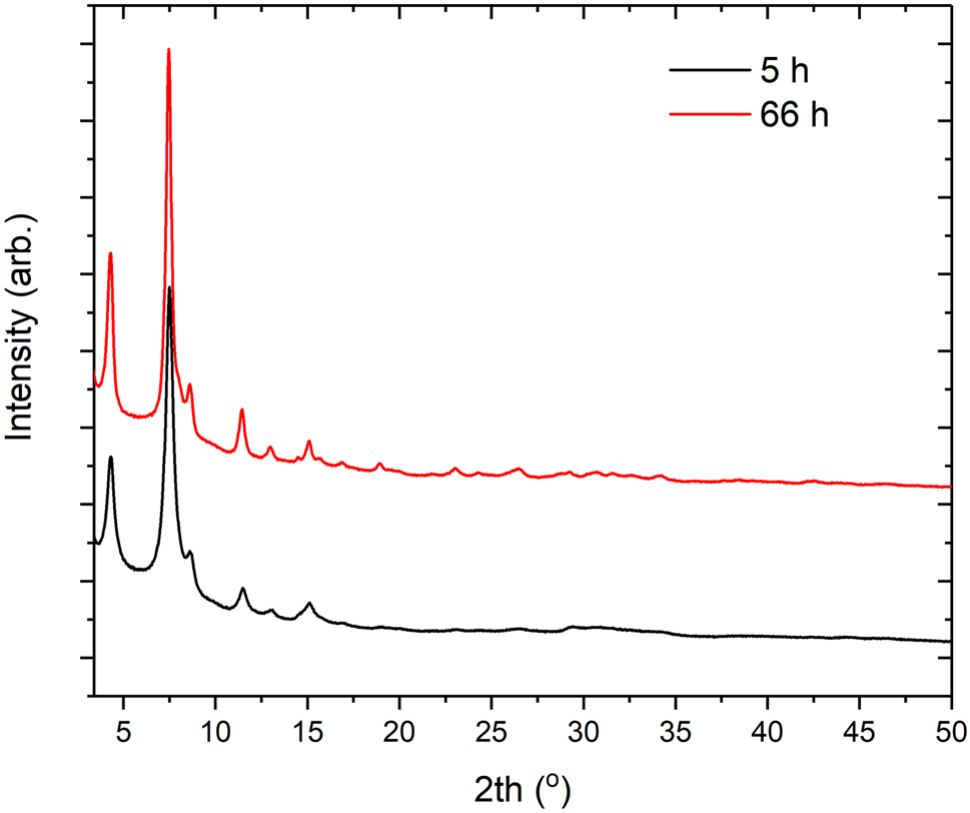
PXRD patterns for Entry 3 produced at 5 and 66 h.

Two small-scale reactions were conducted to determine if material produced at 5 h was comparable to material produced at longer reaction times. The reagent quantities used for these experiments are specified in Table 1 Entry 3. PXRD patterns can be seen in Fig. 6 which show that crystalline material is produced at both short and long reaction times. Importantly the yield of these materials remains constant with the 5 h and 66 h reactions producing yields of 27.08% and 30.31% respectively. Reaction times above 5 h must therefore result in little to no further material production. The increased reaction time does result in sharper peaks observed in the XRD patterns suggesting a growth in crystallite size with time. The crystallite size was determined after Rietveld refinement of the powder patterns using an integral breadth approach, Supplementary Fig. S4 online. Samples produced at 5 h and 66 h have calculated crystallite sizes of 9.854(69) nm and 12.226(87) nm respectively. The increases in diffraction data at higher 2th values indicate more long-range order is present in material produced at longer reaction times. Both the sharper diffraction peaks and the production of little to no extra material being produced is consistent and predicted from the observed plateau in the turbidity data.

**Figure 7:**
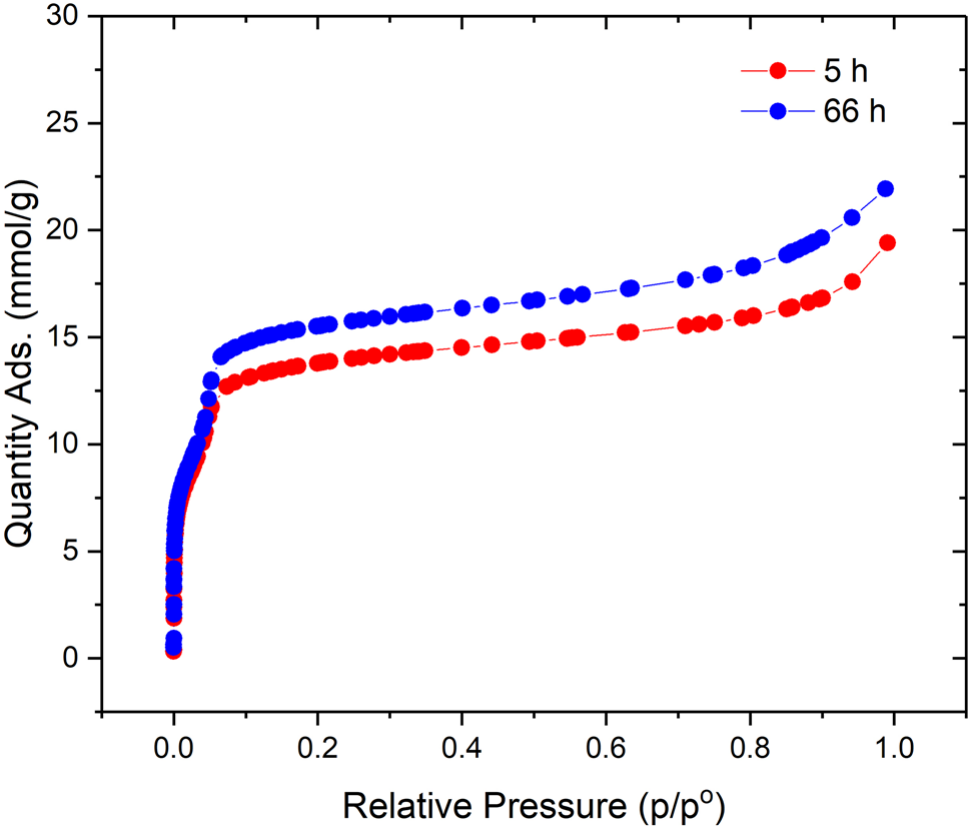
N_2_ isotherms collected on Entry 3 produced at 5 and 66 h.

Physisorption data, Fig. 7, collected on both samples show a difference of just 2 mmol/g at p/p° = 0.1. This increase in uptake may be due to improved long-range order; however, it should not be viewed in isolation. Ultimately these data show that a 13 fold increase in reaction time only produces a 16% improvement in N_2_ uptake. This shows how material produced after 5 h possesses almost all the characteristics needed from this material with only diminishing returns as reaction time increases.

To demonstrate the applicability of this approach in monitoring other MOF preparation routes a zeolitic imidazole framework (ZIF) was prepared and the reaction monitored using the apparatus. ZIFs, a subclass of MOFs, are typified by their zeolite-like structure and have been shown as interesting candidates for a range of application including gas separation and storage^27^. ZIF-8 has been widely studied and consists of tetrahedral Zn^2+^ ions connected by 2-methylimidazole linkers in a porous sodalite topology^28^. Supplementary Fig. S5 online shows turbidity, temperature and pH monitored over the course of a synthesis of ZIF-8. Supplementary Fig. S6 online shows the XRD and Rietveld refinement collected from the material produced during this reaction. It can be seen how a phase pure ZIF-8 material was produced during the reaction.

These data demonstrate changes in turbidity and pH that continue over the 200-min reaction time while the temperature remains constant. These data demonstrate the applicability of the method to other MOF systems, not just STA-16(Ni).

These results show that, after only one experiment with our bespoke, budget data logger, the optimum reaction time for the preparation route of STA-16(Ni) can be predicted. This allowed for the reaction time to be decreased from 72 to 5 h with minimal impact on the quality of the final product—an improvement of 93%. To reach this conclusion previously would have required the removal of sample at various times and collecting and analyzing ex situ XRD patterns. This shows how the in situ monitoring apparatus described in this paper can result in time saving for a very small upfront cost as well as reducing the resources required to reach these conclusions. This key result demonstrates the value of budget in situ reaction monitoring allowing valuable insight to be gained at minimal expense.

## Conclusions

In conclusion we have demonstrated the ability to improve the MOF synthesis route of STA-16 by using budget in situ reaction monitoring. By using a range of sensors, we maximized the chances of observing a reaction critical process that can lead to direct optimization after just one experiment. This is a significant time saving that, in both industry and academia, will result in a sizable advantage within an agile and fast paced research environment. This methodology is applicable to a wide range of other MOF systems as well as other systems where a precipitation reaction occurs, such as the production of metal oxides, zeolites and polymerization reactions. We have demonstrated this by monitoring the production of the MOF ZIF-8. Ultimately, by reaching the conclusions demonstrated above, less time, energy and resources are used to advance chemical science and bring possible products to market.

The reported apparatus is not without its limitations. While calibration is conducted prior to these tests, bespoke instruments lag behind their commercial counterparts which achieve better statistical validation of calibrations^29^. Valuable insight was gained into the STA-16 system and such insight is clearly of benefit to industry scientists working to improve synthetic conditions as well as scientists in academia working at the bench.

Further improvement to this apparatus could include onboard logging via a SD card, reducing the need for a connected computer and Internet of Things modifications could allow this apparatus to be monitored remotely. Additionally, the Arduino® offers many outputs, not just inputs. It is trivial to connect and code pumps or other devices to, for example, add reagents when certain conditions are met, enable samples to be extracted automatically or alter temperature and stirring rates depending on measured experimental variables.

All these processes are currently available in commercial offerings however the ability to quickly prototype and test equipment at the bench can be valuable to research scientists across multiple disciplines and institutions.

## Supplementary information

Supplementary information.
