# Supplementary material for: Use of open source monitoring hardware to improve the production of MOFs: using STA-16(Ni) as a case study
Source: Sci Rep. 2020 Oct 15;10:17355. doi: 10.1038/s41598-020-73780-z (PMC7567096; doi:10.1038/s41598-020-73780-z)
Supplement: Supplementary file 1 — Supplementary information. [file 41598_2020_73780_MOESM1_ESM.docx]

**Use of Open Source Monitoring Hardware to Improve the Production of MOFs: Using STA-16(Ni) as a Case Study.**

Authors: Felicity Massingberd-Mundy^1,2^, Stephen Poulston^1^_,_ Stephen Bennett^1^_,_ Hamish Hei-Man Yeung^2,$^, Timothy Johnson^1*^.

Affiliations:

1 Johnson Matthey Technology Centre, Blount’s Court, Sonning Common, Reading, RG4 9NH, UK.

2 Inorganic Chemistry Laboratory, University of Oxford, South Parks Road, Oxford, OX1 3QR, UK.

$ Current affiliation: School of Chemistry, University of Birmingham, Edgbaston, Birmingham, B15 2TT.

*Correspondence to timothy.johnson@matthey.com

opyright GNU Lesser General Public License


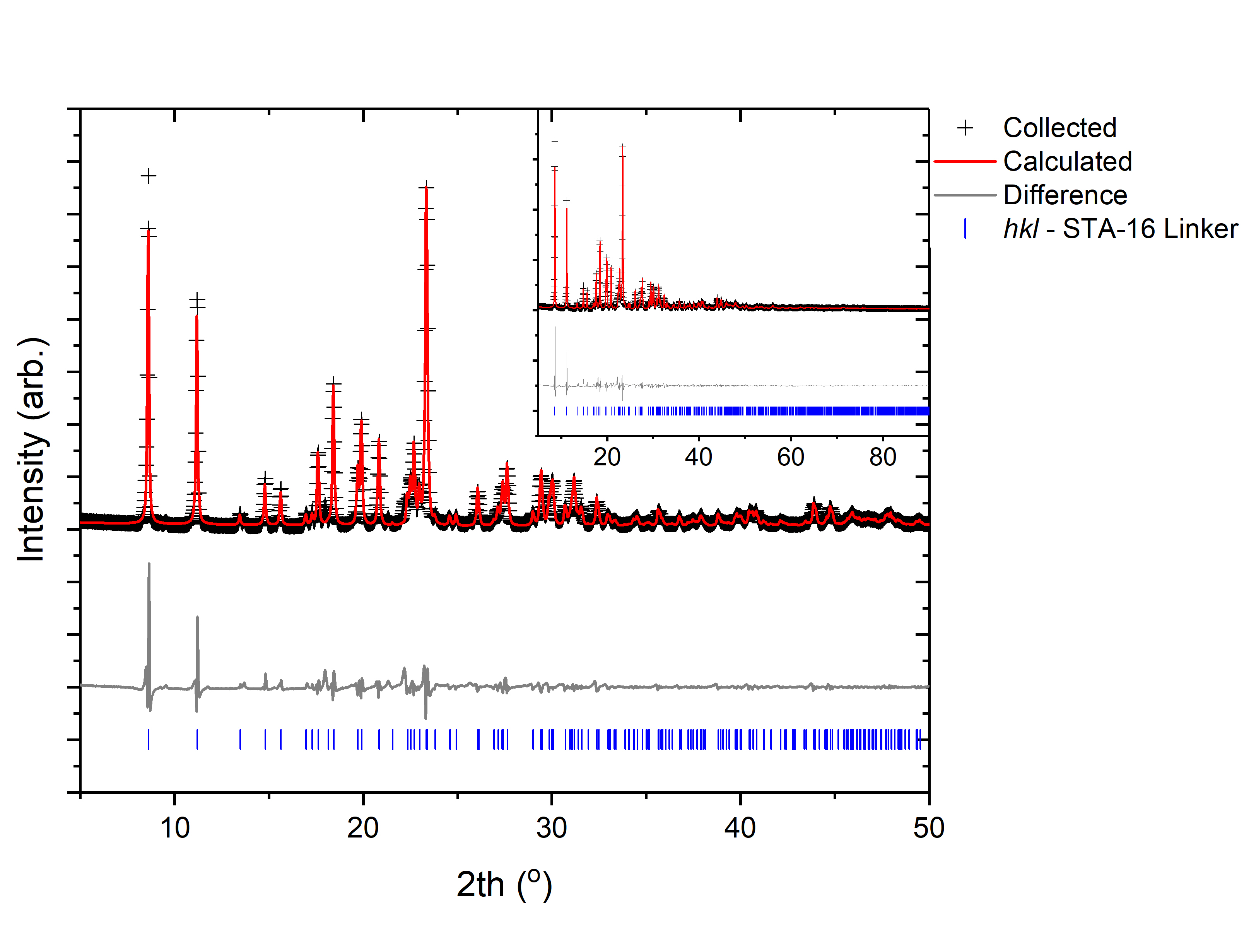


Supplementary Figure S1: PXRD and Rietveld refinement for STA-16 linker.

#include<DFRobotHighTemperatureSensor.h>

#include <Wire.h>

#include "Adafruit_AS726x.h"

unsigned long time;

Adafruit_AS726x ams; //create the object

uint16_t sensorValues[AS726x_NUM_CHANNELS]; //buffer to hold raw values

const float voltageRef = 5.000; //set reference voltage,you need test your IOREF voltage

int HighTemperaturePin = A0; //setting pin for temp

DFRobotHighTemperature PT100 = DFRobotHighTemperature(voltageRef); //Define an PT100 object

#define SensorPin A1 //pH meter Analog output to pin A1

unsigned long int avgValue; //Store the average value of the sensor feedback

float b;

int buf[10],temp;

#define CalibrationGradient 0.2817 //pH calibration constant from gradient of linear trend

#define CalibrationIntercept 4.9544 // pH calibration constant from intercept of linear trend

#define CalibrationConstant 4.1344 //turb calibration constant from A of exponential trend

#define CalibrationExponent -0.0003 //turb calibration constant from exponent of exponential trend

void setup(void) {

Serial.begin(9600); //speed

while(!Serial);

Serial.println(" ");

Serial.print("Time(s)"); //column 1 name

Serial.print(" "); //print tab space

Serial.print("Temp(oC)"); //column 2 name

Serial.print(" "); //print tab space

Serial.print("pH"); //column 3 name

Serial.print(" "); //print tab space

Serial.print("Turb(NTU)"); //column 4 name then start new line

Serial.print(" "); //print tab space

Serial.print("SensorTemp(oC)"); //column 1 name

Serial.print(" "); //print tab space

Serial.print("Violet"); //column 2 name

Serial.print(" "); //print tab space

Serial.print("Blue"); //column 3 name

Serial.print(" "); //print tab space

Serial.print("Green"); //column 4 name then start new line

Serial.print(" "); //print tab space

Serial.print("Yellow"); //column 2 name

Serial.print(" "); //print tab space

Serial.print("Orange"); //column 3 name

Serial.print(" "); //print tab space

Serial.println("Red"); //column 4 name then start new line

}

void loop(void) {

time = millis(); //get time

float seconds = time/1000; //convert to seconds

Serial.print(seconds); //print time

Serial.print(" "); //print tab space

pinMode(13,OUTPUT);

int temperature = PT100.readTemperature(HighTemperaturePin); //get temp

Serial.print(temperature); //print temp

Serial.print(" "); //print tab space

for(int i=0;i<10;i++){

buf[i]=analogRead(SensorPin);

delay(10);

}

//each time i is smaller than 10, read sensor, then increase i by 1

//so eventually 10 readings are collected (i=0, 1, 2, 3, 4, 5, 6, 7, 8, 9)

for(int i=0;i<9;i++){

for(int j=i+1;j<10;j++){

if(buf[i]>buf[j])

{

temp=buf[i];

buf[i]=buf[j];

buf[j]=temp;

}

}

}

//j is the next consecutive i, so if the reading from i is bigger than the reading from j, the reading from i and the reading from j swap

//so organise the 10 readings from smallest to largest value

avgValue=0;

for(int i=2;i<8;i++)

avgValue+=buf[i];

//each time i=2, 3, 4, 5, 6, 7, add the reading to the avgValue.

//so get the total of the 6 middle values

float phValue=(float)avgValue*5.0/1024/6; //convert the total into a voltage and divide by 6 to get the average

phValue=3.5*phValue; //convert the average voltage into pH value

phValue=phValue-CalibrationIntercept;

phValue=phValue/CalibrationGradient; //calibration

Serial.print(phValue,2); //print pH reading to 2.d.p

Serial.print(" ");

digitalWrite(13, HIGH); //sets digital pin voltage to 5V

digitalWrite(13, LOW); //sets digital pin voltage to 0V

int sensorValue = analogRead(A2);// read the input on pin A2, resistance value 0-1023

float voltage = sensorValue * (5.0 / 1024.0); // Convert the analog reading (which goes from 0 - 1023) to a voltage (0 - 5V):

double turbiditycalc = voltage/CalibrationConstant;

double turbidity = (log(turbiditycalc))/CalibrationExponent; //calibration and conversion from volts to NTUs

Serial.print(turbidity); // print turb

Serial.println(" "); //print tab space then start new line

// initialize digital pin LED_BUILTIN as an output.

pinMode(LED_BUILTIN, OUTPUT);

//read the device temperature

uint8_t temp = ams.readTemperature();

ams.drvOn(); //uncomment this if you want to use the driver LED for readings

ams.startMeasurement(); //begin a measurement

//wait till data is available

bool rdy = false;

while(!rdy){

delay(5);

rdy = ams.dataReady();

}

ams.drvOff(); //uncomment this if you want to use the driver LED for readings

//read the values!

ams.readRawValues(sensorValues);

//ams.readCalibratedValues(calibratedValues);

Serial.print(temp);

Serial.print(" ");

Serial.print(sensorValues[AS726x_VIOLET]);

Serial.print(" ");

Serial.print(sensorValues[AS726x_BLUE]);

Serial.print(" ");

Serial.print(sensorValues[AS726x_GREEN]);

Serial.print(" ");

Serial.print(sensorValues[AS726x_YELLOW]);

Serial.print(" ");

Serial.print(sensorValues[AS726x_ORANGE]);

Serial.print(" ");

Serial.print(sensorValues[AS726x_RED]);

Serial.println(" ");

delay(800); //slow down output so easier to read

}

Supplementary Figure S2: Code used for the in-situ reaction monitoring apparatus. Code taken from various manufacturers of probes and combined above. Please see Table 2


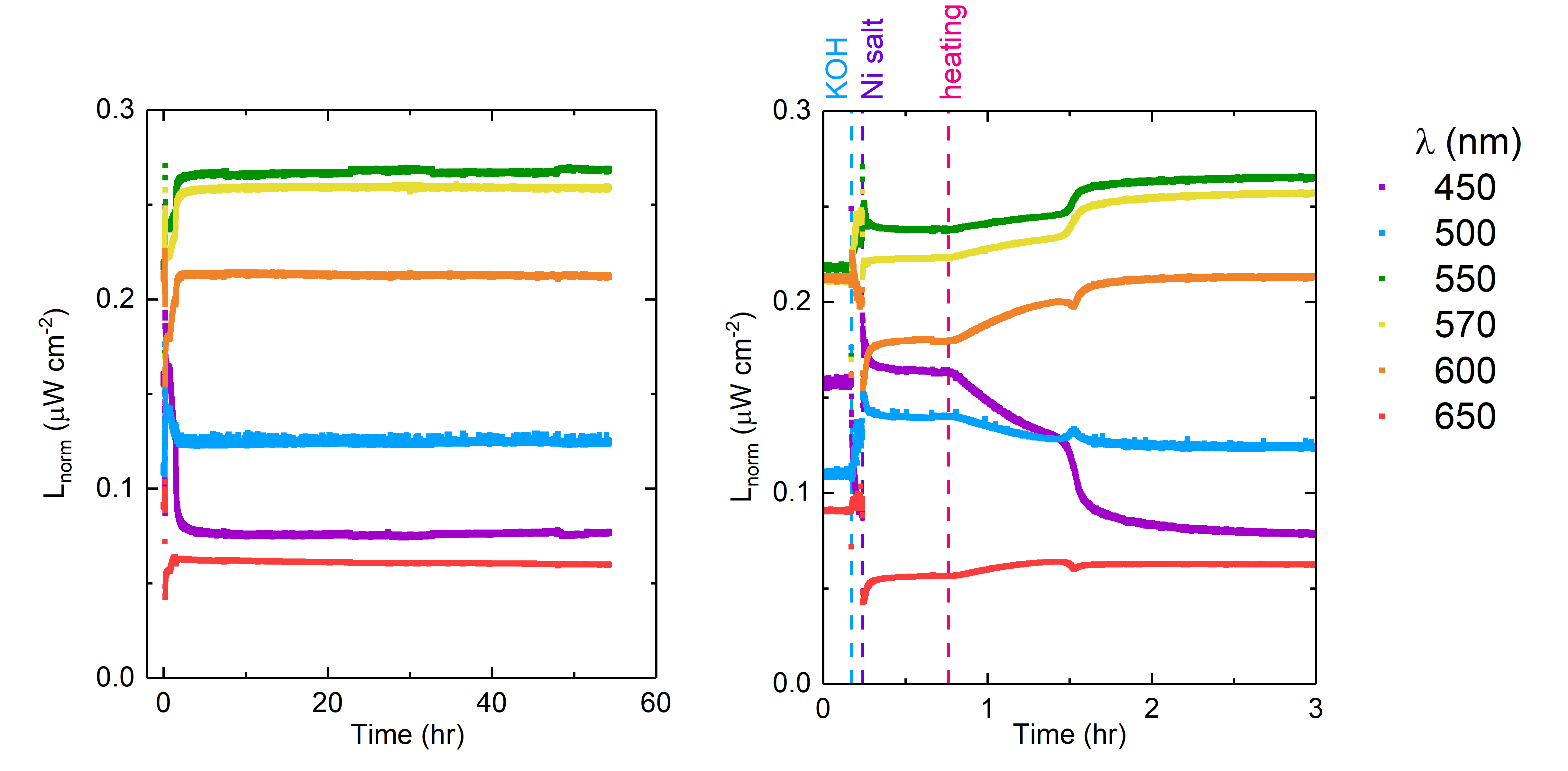


Supplementary Figure S3: left) pH data collected during the preparation of STA-16(Ni), right) collected in-situ spectra at various wavelengths.


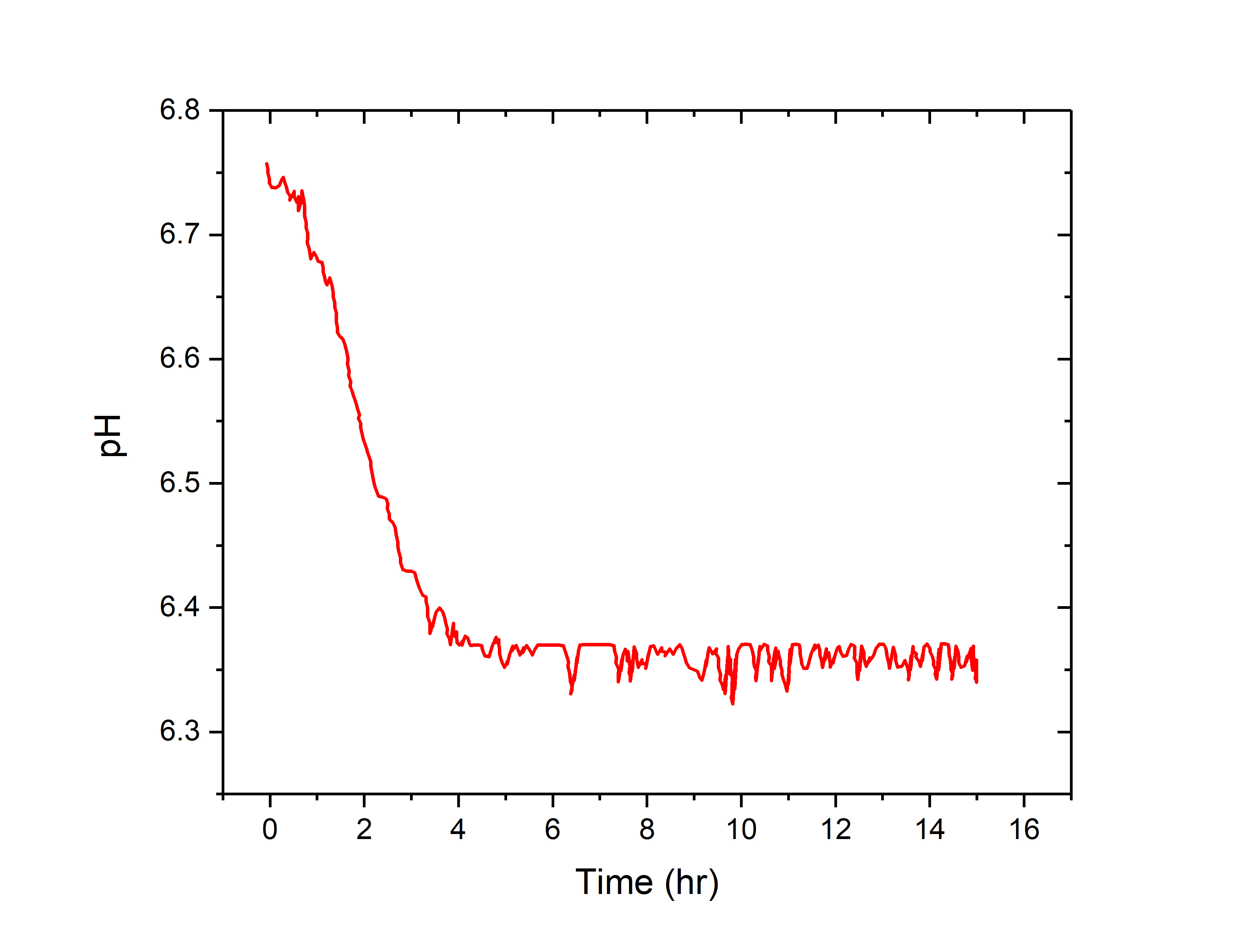


Supplementary Figure S4: PXRD patterns and Rietveld refinements for Entry 3 (see table 1 in main text for reagent quantities) produced at 5 and 66 hours respectively.


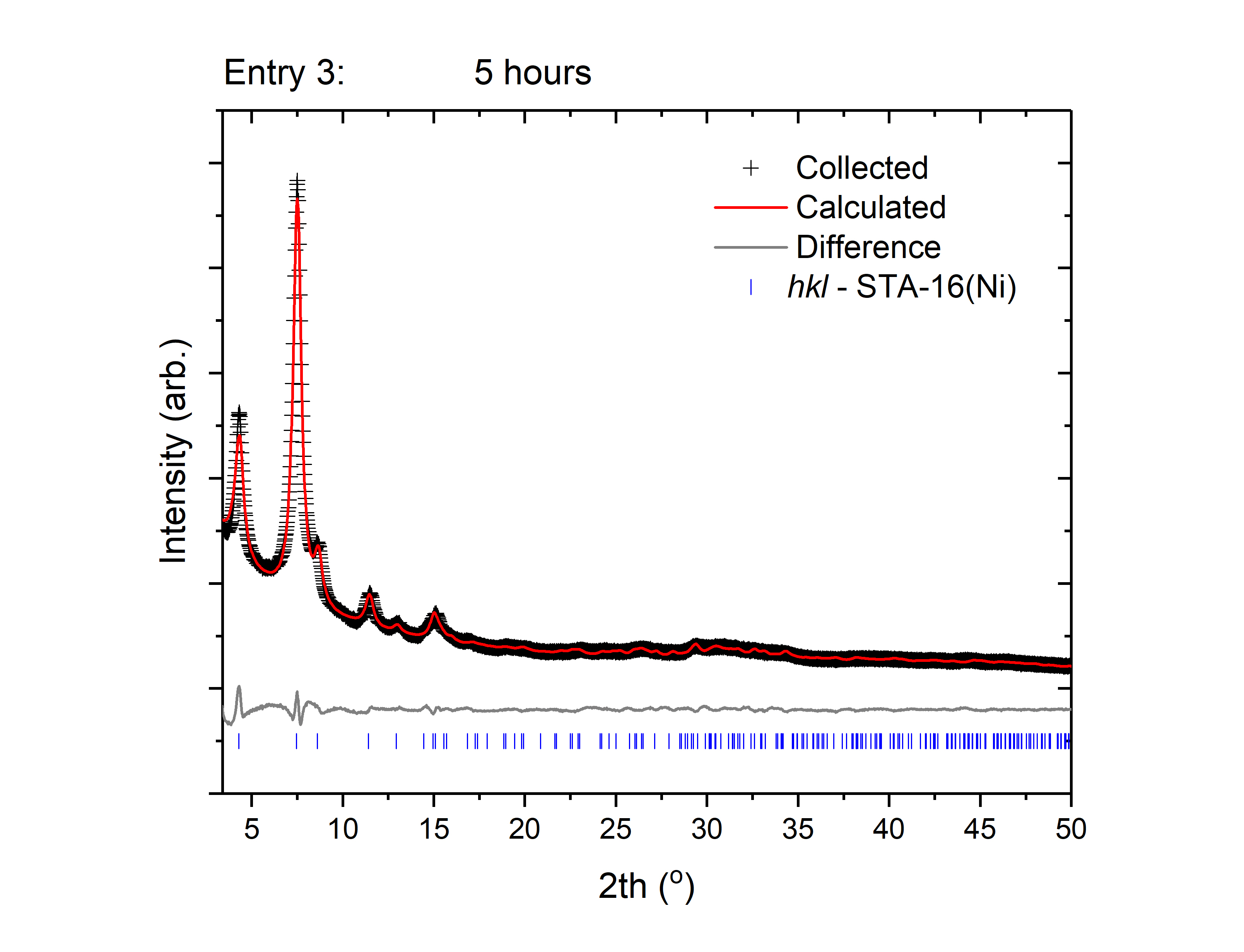

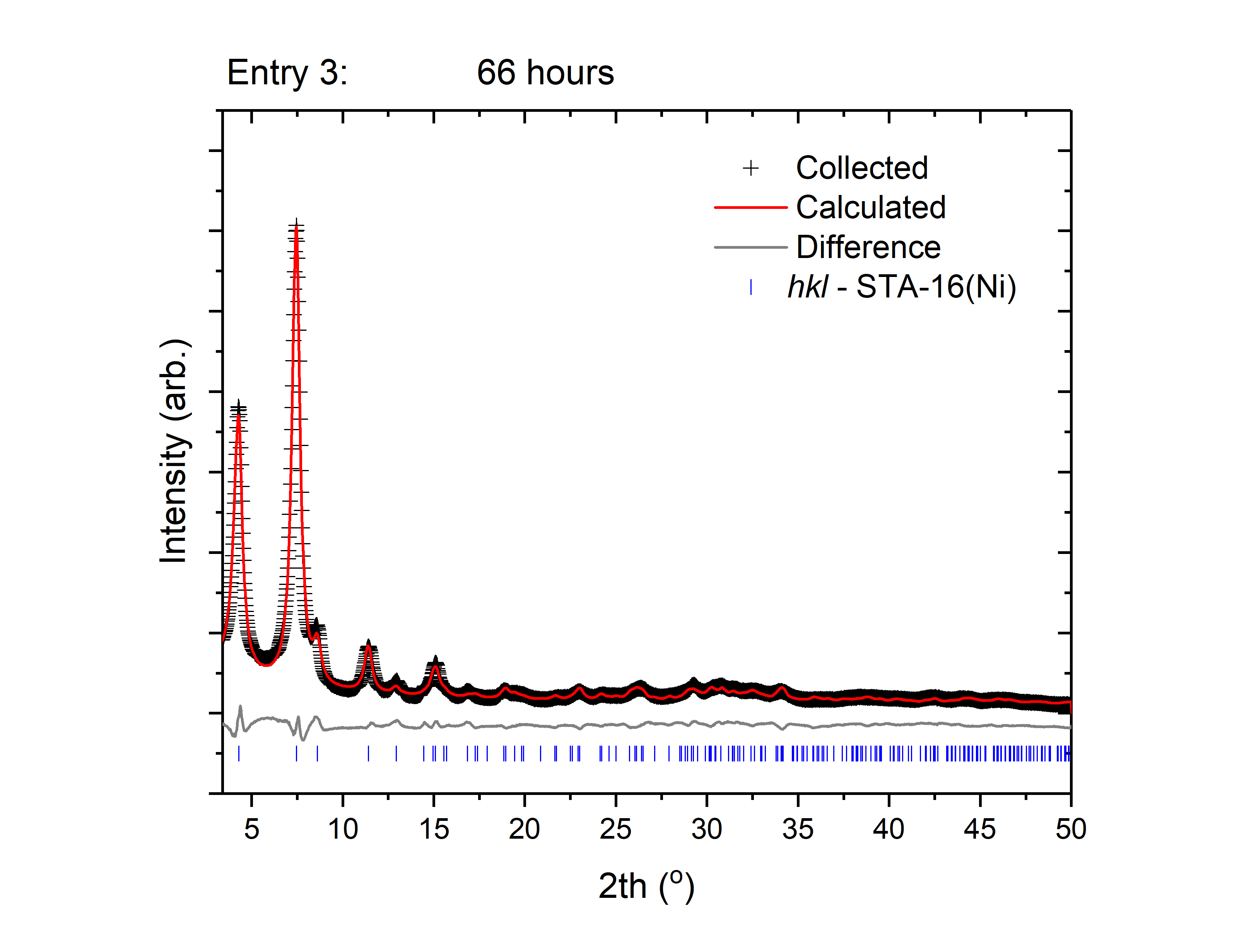


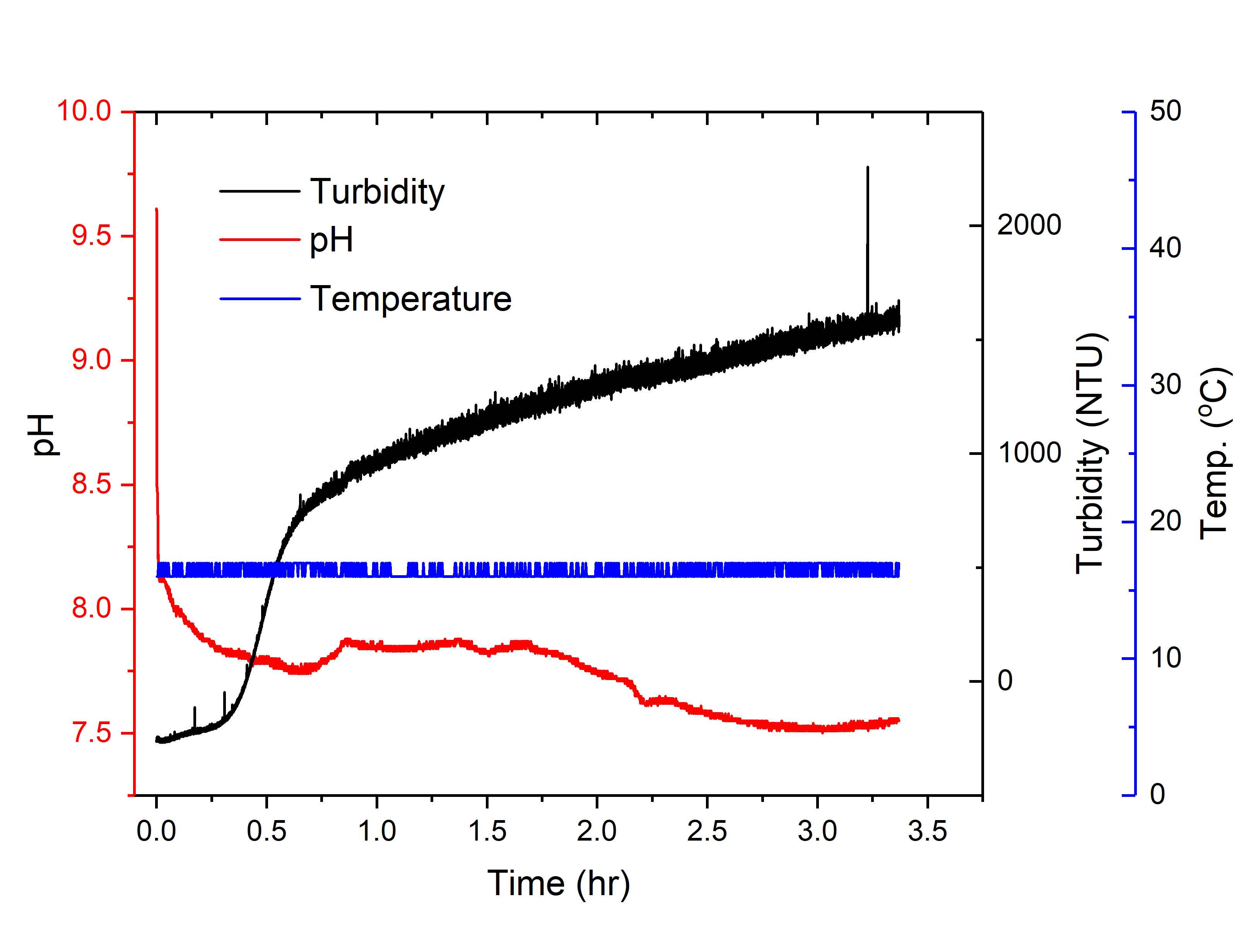


Supplementary Figure S5: Turbidity, pH and temprature data collected during the production of ZIF-8.


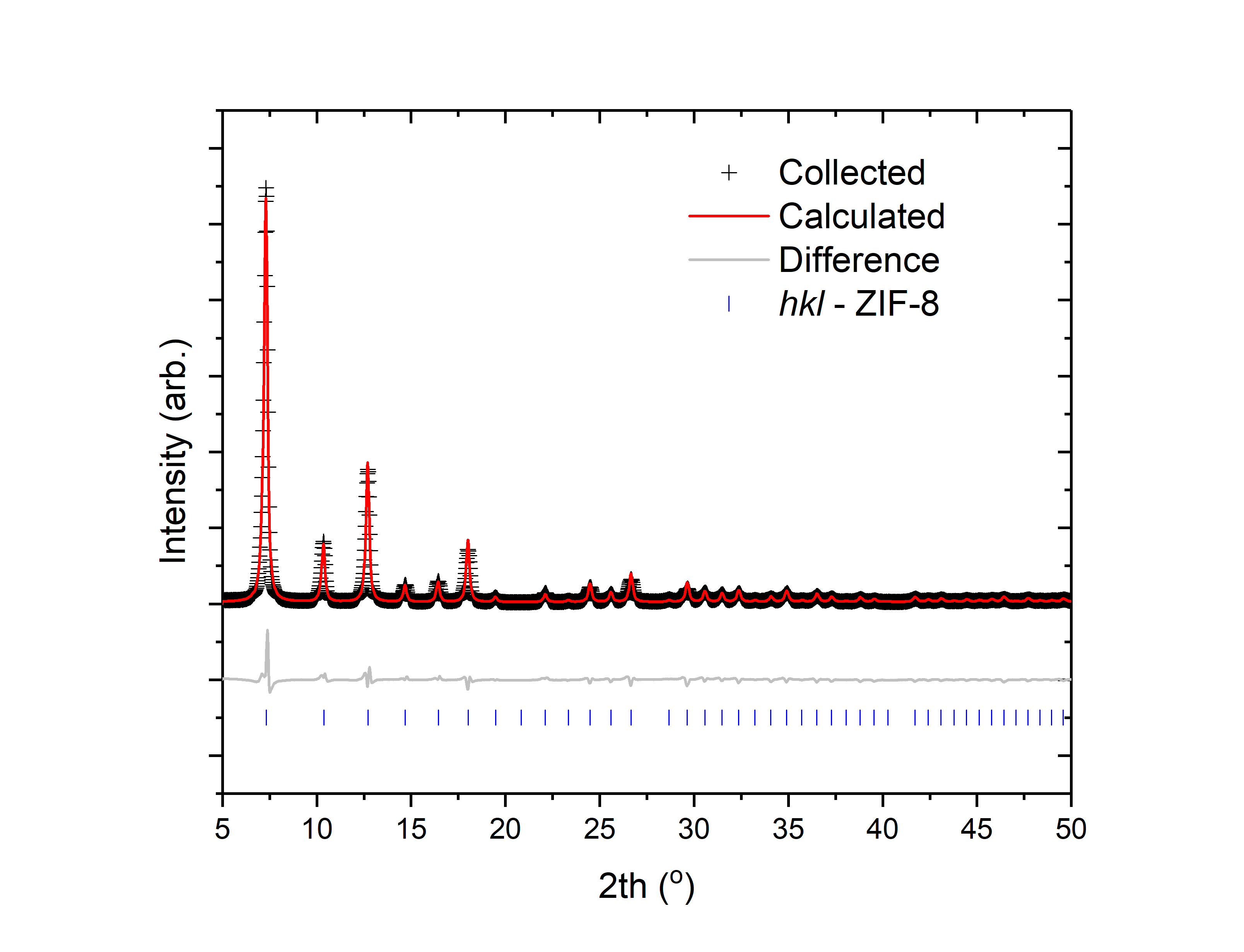


Supplementary Figure S6: PXRD and Rietveld refinement of ZIF-8.
